# Extracellular vesicles as vaccine platforms: emerging opportunities for malaria

**DOI:** 10.3389/fimmu.2026.1870149

**Published:** 2026-07-06

**Authors:** Gisele Tatiane Soares da Veiga, Jordana Dinorá de Lima, Letusa Albrecht

**Affiliations:** Instituto Carlos Chagas, Fundação Oswaldo Cruz, Curitiba, Paraná, Brazil

**Keywords:** engineered EVs, malaria, neglected diseases, *Plasmodium*, prevention

## Abstract

Malaria is the deadliest parasitic disease worldwide, and the urgent need for preventive strategies remains unmet. The parasite poses unique challenges, as it exhibits remarkable epitope variability and undergoes genetic mutations that allow adaptation to control measures, including drug treatment, and high ability of immune system evasion. In addition, malaria frequently develops asymptomatic forms, which sustain silent transmission and perpetuate the disease burden. In this complex context, extracellular vesicles (EVs) have emerged as promising tools for vaccine development. EVs can be engineered to carry specific and various antigens and to exploit their natural ability to fuse with cell membranes, enabling effective delivery. Depending on their cargo, they can modulate immune responses in a tailored manner, reawakening host immunity and targeting the parasite even in dormant stages. Despite extensive advances in EV-based approaches for viral and cancer models, their application to neglected diseases, including malaria, remains limited. This review aims to discuss strategies for engineering EVs as vaccines, drawing on insights from other disease models and highlighting the unique features of malaria that could benefit from such an approach.

## Introduction

The increase in vaccination rates is closely linked to the reduction in morbidity and mortality caused by infectious diseases, according to data from the World Health Organization. Despite these advances, considerable challenges remain in preventing diseases for which safe and effective vaccines are not yet available (1). Notable among these are neglected infectious diseases (NTDs) which disproportionately affect socially and economically vulnerable populations (2). These diseases are historically marked by low investment in research and development, which limits access to adequate prophylactic therapies.

Among neglected tropical diseases (NTDs), malaria stands out due to its complex life cycle, which includes at least three distinct stages: pre-erythrocytic, erythrocytic, and sexual in the invertebrate host (3, 4). Recently, significant advances have been made in vaccine development, with RTS,S and R21 emerging as the first candidates to achieve approval (5, 6). Both vaccines target the same antigen, the circumsporozoite protein (CSP), which plays a crucial role in sporozoite proliferation within the mammalian host. This redundancy highlights the urgent need for alternative vaccine targets, particularly given the remarkable epitope variability exhibited by *Plasmodium* species. Such variability has already posed challenges in pharmacological interventions, as evidenced by the growing resistance of *P. falciparum* to artemisinin (7–9). This resistance has necessitated the use of combined therapies with secondary drugs, which now provide alternative strategies for malaria treatment (10). Drawing from this experience, vaccine development must also evolve to diversify its targets, ensuring broader efficacy and resilience against parasite variability.

Conventional types of vaccines include usage of proteins, peptides, or pathogens – either attenuated or inactivated - while genetic-based vaccines could implement adenoviral, retroviral platforms, as well as the recent self and trans-amplifying RNA (11). Alongside these advances, extracellular vesicles (EVs) have emerged as a promising and cost-effective platform (12), particularly due to their ability to stabilize carried molecules and extend the half-life of cargos such as RNA (13), to allow for possibilities of genetic modifications in the producing cells (14), insertion of molecules of interest onto the surface of the vesicles (15, 16), or loading antigens (17–19) and adjuvants (20) directly onto the isolated EVs. EV engineering therefore aims to increase their stability, specificity, and immunogenicity (21, 22), making them even more attractive vaccine platforms and potential alternatives in the fight against neglected diseases.

However, even though it is promising, the use of EVs for a malaria vaccine remains underexplored. In this context, this paper aims to present a critical review of extracellular vesicle engineering strategies applied to vaccination, with an emphasis on their use in combating malaria. By gathering recent evidence, we discuss technological advances, ongoing challenges, and prospects for the implementation of modified EVs as innovative and affordable alternatives to combat these historically neglected diseases.

## Literature search strategy

This narrative review explores the intersection of EVs, EV engineering, vaccine platforms, and malaria. A comprehensive literature search was executed in the PubMed, Scopus, and Web of Science databases for English-language original articles and reviews published up to April 2026. The search strategy employed terms such as “extracellular vesicles”, “exosomes”, “engineered EVs”, “malaria vaccine”, “*Plasmodium*”, “vaccine delivery”, and “immune modulation”, used individually or in combination. The selection process prioritized studies focusing on EV-based therapeutic strategies, cargo engineering, mechanisms of immune activation, and applications in infectious diseases or cancer. To ensure coverage, the bibliographies of the selected articles were also manually screened for additional relevant references.

## EVs structure and characterization

EVs are lipid bilayer–enclosed nanoparticles that cannot be self–replicated (23). They are produced through two main pathways: they can bud directly from the plasma membrane (micro vesicles) or originate from intracellular compartments known as multivesicular bodies (exosomes) (23).

EVs mediate intercellular communication by transferring biomolecules such as proteins, lipids, RNAs, and other signaling molecules (24). Through these interactions, they participate in essential homeostatic processes, including the clearance of unwanted cellular components, regulation of cell maturation and activation, tissue repair and regeneration, blood coagulation, and adaptation to environmental changes. They are also implicated in pathological contexts such as tumorigenesis and neurodegeneration (25–27). Importantly, EVs can modulate immune responses by delivering bioactive cargo to specific target cells (28).

Due to their natural role as molecular carriers, EVs have attracted attention as promising platforms for therapeutic delivery (29). Their nanometric size, low immunogenicity, minimal cytotoxicity, and ability to efficiently cross biological barriers (30–33) make them safe for long-term use. Furthermore, their biological origin provides enhanced stability, optimized biodistribution, and a lower risk of uncontrolled immune activation (34). Taken together, these features position EVs as a versatile and powerful tool for the development of innovative strategies, including those aimed at managing infectious diseases.

## EVs mechanisms of immune activation

**Figure 1:**
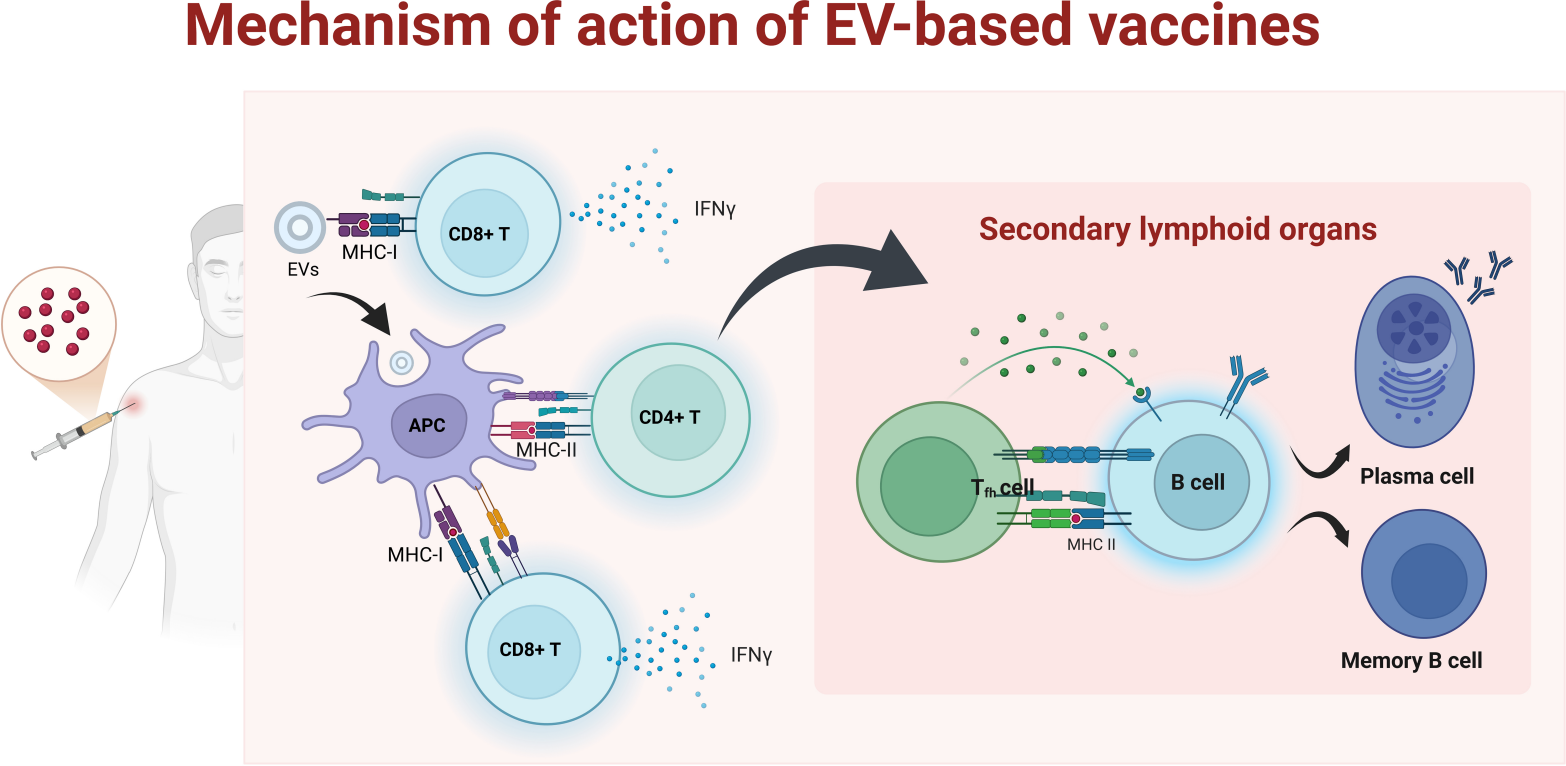
Schematic representation of main immune activation of EVs. EVs can stimulate CD8+ T or antigen-presenting cells (APCs), leading to the activation of CD4^+^ and CD8^+^ T cells and the release of IFN-γ. Activated CD4^+^ T helper cells subsequently interact with B cells in secondary lymphoid organs, promoting their differentiation into plasma cells and memory B cells, generating immunological memory. EV, extracellular vesicle; APC, antigen-presenting cell; MHC, major histocompatibility complex; Tfh cell, T follicular helper cells. Created in BioRender. Albrecht, L. (2026). https://BioRender.com/fz8mnsc.

Extracellular vesicles (EVs) play important roles in regulating the immune response, primarily acting on humoral immunity (35), although they also interfere with cellular immunity (36, 37) (**Figure 1**). Studies in cancer models have shown that EVs can reduce cell migration (38) and tumor growth (39, 40), promote tumoricidal responses (36), and favor the infiltration of immune cells into the tumor microenvironment (36). Similarly, in infectious disease settings, EVs have been shown to reduce viral infection capacity (41) and increase viral neutralization (42), in addition to stimulating the activation and proliferation of T cells (36, 37).

Several organs and tissues are involved in EV-mediated immune activation. The liver (43), splenocytes (44), and spleen (17) are prominent in this process, as are the lungs (17), reflecting the broad role of EVs in adaptive immunity. In these regions, there is evidence that EVs modulate B cell activity (45) and promote the expression of Human Leukocyte Antigen (HLA) (46) and (Major Histocompatibility Complex class I (MHC I) (47–49) molecules, essential for antigen recognition and antigen presentation.

Regarding immune cells and mediators, the EV administration induces the production of immunoglobulins, such as IgG (13, 50–52), IgM (20, 51), and IgA (44, 50, 51) antibodies. This production is primarily through the activation of CD4+ (47, 50, 53) and CD8+ (18, 37, 49, 50) T cells. Furthermore, EVs stimulate the production of IFN-γ by CD8+ cells (48) and promote the release of cytokines associated with a Th1 profile (50), enhancing the antiviral and antitumor response capacity. These findings indicate that EVs function as versatile modulators of the immune response, integrating humoral and cellular signals to promote protection against pathogens and tumor cells.

## Cellular immunity

The cellular response to antigens can initially occur mediated by antigen-presenting cells (APCs) together with the participation of EVs. As an example, APCs exposed to chemically inactivated Foot and Mouth Disease Virus (FMDV) were able to produce EVs containing proteins and peptides of the virus. The EVs produced by the stimulated APCs were useful in the activation of B and T cells, especially those from marginal zone B cells (MZB) and follicular B cells (FoB) in the spleen (45).

Naturally, vesicles are important carriers of information. Exploiting this ability, mice were exposed to EVs containing the SARS-CoV-2 nucleocapsid protein fused to Nef^mut^ —highly enriched in vesicles—after which the animals were exposed to the viruses themselves and survived (19). The response, in this case, was attributed to the activation of CD8+ T cells (19).

In addition to this primary stimulus, EVs can also act by increasing the intensity of the cellular response. For example, peripheral blood mononuclear cells (PBMC) from individuals already vaccinated against SARS-CoV-2 were exposed to EVs containing the spike protein fused to the PDGFRβ transmembrane domain and were able not only to internalize it but also to activate CD4+ and CD8+ T cells after administration of these EVs (37).

The boost resulting from exposure to EVs can also occur through the production of chemokines, facilitating the response to the antigen. In this case, studies have described that EVs could be useful in increasing CD4+ and CD8+ T cell responses after administration of DNA vaccines (20). A similar phenomenon occurred with prior exposure to adenovirus vaccines (35). These results highlight the potential of EVs to efficiently modulate adaptive immune responses.

## Humoral immunity

EVs play a central role in antigen presentation, promoting antigen-specific immune responses and the development of long-term immunological memory (28). By carrying antigens or immune-modulating molecules, they can enhance both antigen processing and presentation by immune cells (28). Antigen presentation may occur directly, through MHC molecules displayed on the EV surface—often accompanied by costimulatory molecules—which enable stimulation of T cells (28, 54, 55).

EVs may also mediate indirect presentation, being internalized by antigen-presenting cells (APCs), such as dendritic cells (DCs), which process EV-associated antigens. Processed peptides are then presented on MHC II molecules to CD4+ T cells in secondary lymphoid organs (47, 56). EVs can also interact with APCs in ways that facilitate B cell activation (56). Moreover, DCs can acquire EVs carrying peptide–MHC I complexes in a process known as cross-presentation, leading to the priming of naïve CD8+ T cells (49). If EVs are engineered to carry mRNA, the transcript can be translated into protein within recipient cells and presented via MHC I to CD8+ T cells (13, 57). Alternatively, the newly synthesized protein may be endocytosed by APCs, processed, and presented through MHC II to CD4+ T cells in a conventional manner (13, 57).

## EVs engineering strategies

### Origin and composition of engineered EV

Virtually all cell types can produce extracellular vesicles (EVs), although the intrinsic properties of each cell influence both vesicle yield and composition. Among the most widely used sources in EV engineering studies are mammalian cells, especially immortalized cell lines. Cell lines such as HEK 293T (17, 18, 21, 22, 36, 41–44, 52, 58, 59), or the 293F (13, 20, 35, 37) are chosen for producing the EVs due to the ease of production. To a lesser extent, Chinese hamster ovary (CHO) cells (60) have also been used, as their EV release is considered superior. Primary cell lines, such as isolated dendritic cells (DCs) or lab engineered lung spheroid cells (LSCs), can also be used, albeit with limitations regarding their availability and biological variability. In addition to mammalian cells, unconventional sources have been explored as plant cells (61) and EVs. These alternative sources offer unique insights into molecular composition and immunogenicity, although they still require optimization for therapeutic applications.

**Figure 2:**
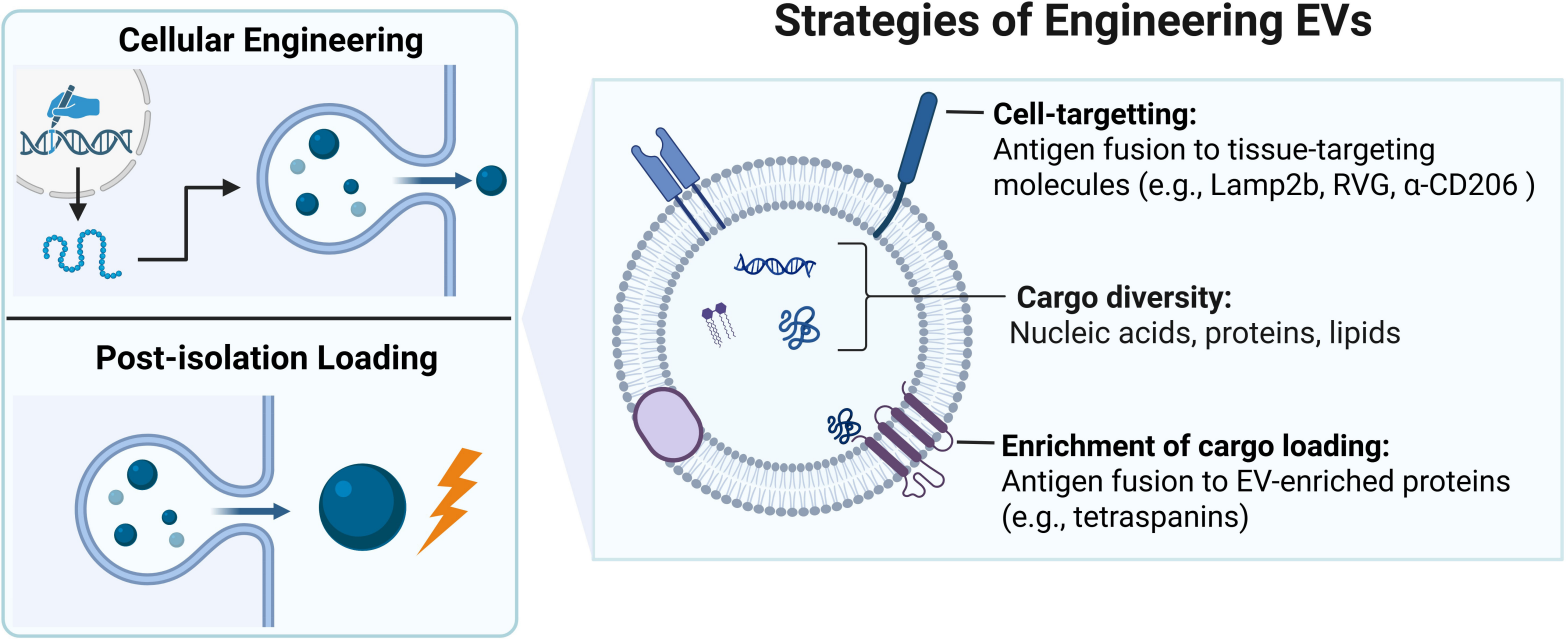
Strategies for engineering extracellular vesicles (EVs). EVs can be engineered either by *cellular engineering*—modifying parental cells to express or package specific molecules—or by *post-isolation loading*, which introduces cargo into isolated EVs through physical or chemical methods. Engineering strategies may aim at (i) cell targeting, by fusing antigens to tissue-specific molecules (e.g., Lamp2b, RVG, α-CD206); (ii) cargo diversification, including nucleic acids, proteins, and lipids; and (iii) cargo loading enrichment, through fusion of antigens to EV-enriched proteins such as tetraspanins. Lamp2b: lysosome-associated membrane glycoprotein 2 isoform b, RVG, rabies virus glycoprotein; α-CD206, anti-CD206. Created in BioRender. Albrecht, L. (2026). https://BioRender.com/qltj2co.

EV engineering depends on both cellular origin and cargo insertion strategies. In mammalian systems, techniques such as protein overexpression can increase EVs production and promote the incorporation of specific proteins (62), expanding the potential of EVs as natural delivery vehicles (63) (**Figure 2**). Incorporation methods include EVs electroporation (64), nanoporation (39), viral transduction (13) and transfection of parental cells (60, 65), or even direct transfection of exosomes (46), allow the loading of additional molecules, such as messenger RNA, peptides, or proteins, significantly expanding EVs functionality.

In general, the choice of cell source and cargo insertion method must be carefully considered, as it affects not only production and encapsulation efficiency but also the biochemical and immunological composition of EVs, determining their potential for therapeutic and immune targeting applications.

### For cell-targetting

The use of EVs allows for specific targeting of tissues or cell types, expanding their potential as therapeutic vehicles. For this targeting to be effective, the proteins present on the surface of EVs must be selectively recognized by the target tissue or cell, and this hypothesis has been validated experimentally. To illustrate this phenomenon, by exploiting the ligand-receptor principle, EVs were targeted to cardiac tissue through the incorporation of Lamp2b, a peptide recognized specifically by muscle cells (66). Targeting can also be applied to neural tissues. EVs containing Rabies Virus Glycoprotein (RVG)—a small protein derived from the rabies virus glycoprotein that binds to acetylcholine receptors on neuronal cells—have shown the ability to deliver content specifically to cells of the nervous system (59).

Furthermore, immune cells can be specifically targeted by EVs. For example, macrophages were targeted with EVs containing α-CD206 antibodies, ensuring selective delivery to this cell type (22). Similarly, exosomes containing the H-Y-I-Ab protein complexes – a protein from Y cromossome bound to an MHC I complex - were recognized exclusively by T cells from female Marilyn mice as non-self-antigen (47).

These studies demonstrate that EV targeting can be achieved through the appropriate selection of surface proteins or specific ligands, allowing vesicles to be used for highly precise therapeutic applications, both in specific tissues and in cell subpopulations.

### For enrichment of cargo loading

However, some proteins are not naturally present inside the EVs, so, there is a need for enriching the content of the EV. Cargo enrichment is a central strategy in the engineering of EVs, enabling the controlled incorporation of proteins, antigens, or therapeutic molecules. Certain viral transmembrane domains and proteins naturally associated with EVs, such as Nef ^mut^ antigen (17, 18, 46), CD81 (60), or CD63 (20), can be used as scaffolds for the binding of other proteins, facilitating their incorporation into the EVs. Scaffolds can, for example, bind to DNA vectors (18) or viral antigens (17), expanding the delivery potential and immunogenicity of EVs.

The modularity of these approaches allows the construction of EV fragments capable of binding proteins interchangeably, enabling rapid optimization for different antigens. A demonstrable example of this concept was the engineering of an EV containing incomplete GFP (60); by adding the missing fragment of the molecule (GFPs11) attached to a protein of interest, that protein could be enriched in the EV. Similarly, the CD9-HUR complex can be used to bind DNA molecules to vesicles (43), further expanding the possibilities for functional cargo.

Furthermore, the position of the protein of interest within the EV can be targeted according to the desired signaling. For example, fusion with brain acid-soluble protein 1 (BASP1) directs the protein to the interior of the EV, while fusion with Prostaglandin F2 Receptor Negative Regulator (PTGFRN) exposes it on the external face of the EV (15, 40, 52). Other proteins, such as MHC-I, lactaderin (C1C2 domain), and several tetraspanins, can also be used to enrich or modulate vesicle cargo, offering a versatile repertoire of tools for engineering EVs with therapeutic or immunological applications (**Table 1**).

**Table 1:** Summary of the main scaffold proteins for EVs cargo enrichment.

EV donor cell	Scaffold protein	Reference
BMDCs	CD47	(39)
CHO	CD81	(60)
DC	Lamp2b	(64)
Neuro2A cells	GPI anchor	(67)
WEHI	Lactadherin	(46)
Muscle cells	Nef^mut^ protein	(17, 18, 46)
HEK293	Lamp2b	(66)
HEK293T	CD47	(68)
HEK293T	CD9	(43)
HEK-293T	TSPAN2, TSPAN3, CD63	(69)
HEK293	CD63	(15, 41, 52)
HEK293	CD63, CD81, VSV-G	(70)
HEK293	PTGFRN, BASP1	(40)
HEK293T	CD9	(42)
HEK293T	VSV-G	(58)
HEK293T	LEAP	(36)
293F	PDGFRβ	(37)
HEK293T	PTGFRN-Δ687	(59)

BMDC, bone marrow-derived cells; CHO, Chinese hamster ovary cells; DC, dendritic cells; HEK, human embryonic kidney cells; Lamp2b, lysosomal-associated membrane protein 2; GPI, glycosylphosphatidylinositol; Nefmut, mutated Nef protein; TSPAN, tetraspanins; PTGFRN, prostaglandin F2 receptor negative regulator; VSV-G, envelope glycoprotein of the vesicular stomatitis virus; LEAP, liver-expressed antimicrobial peptide; PDGFR, platelet-derived growth factor receptor.

In the context of malaria, these engineering strategies may enable distinct antigen presentation approaches according to parasite biology. Surface display of antigens through scaffold proteins such as PTGFRN may be particularly advantageous for highly polymorphic parasite proteins, including CSP or MSP family members, by promoting multivalent antigen presentation and increasing antigen density on the EV surface, which may enhance immune recognition and B-cell activation. In contrast, internal encapsulation strategies may protect conserved intracellular antigens or nucleic acid cargos from degradation, potentially improving stability and delivery efficiency.

### Cargo diversity

A variety of molecules can be enclosed by an EV with the final goal of modulating the immune system. The most common are proteins, DNA and RNA. As an example of trapping proteins, SARS-Cov peptides (S1, S2, M, or N) were fused to the Nef^mut^ protein using pVAX vector, leading to the production of modified EVs in mice immunized (17). This process resulted in the activation of CD4+ T cells and, more prominently, CD8+ T cells, with responses observed in both the spleen and lung airways (17).

Alternatively, EVs can be targeted with DNA directly. DNA vectors-containing EVs proved highly effective in eliciting antigen-specific cytotoxic CD8+ T cell responses against HPV, Ebola, Influenza, and Hepatitis C in mice immunization (18). Also, EVs represent an effective method for delivering mRNA. Based on that, Pomatto et al. (61) utilized orange juice (*Citrus sinensis*) EVs to encapsulate and deliver SARS-CoV-2 mRNA (61). EVs effectively safeguarded the mRNA from degradation and ensured efficient delivery to APCs, promoting TCD4+ cell proliferation (61). Additionally, oral and intranasal delivery methods outperformed the intramuscular route by providing robust mucosal IgA protection and enhancing the activation of splenic immune cells (61).

In a complementary way, another approach involves utilizing a recombinant protein overexpression system to encapsulate both mRNA and proteins within EVs. Luo et al. (13) revealed that EVs containing the mRNA and Spike protein effectively induced the production of neutralizing IgG antibodies and antigen-specific T cell responses, particularly CD4+ T cells, in both mice and baboons (13). Similarly, Ferrantelli et al. (17) demonstrated that different antigens carried by EVs can be cross-presented to effectively activate CD8+ T cells (17). These findings underscore the potential of EVs as a versatile platform for developing multiantigen vaccines against a wide range of pathogens, including promising applications for malaria control.

## Perspectives and application of EV vaccines for malaria

Malaria is a serious disease, responsible for 263 million cases and more than 590,000 deaths annually. The species *Plasmodium falciparum* was responsible for 95% of malaria deaths worldwide in 2023 (71), while *Plasmodium vivax* represents approximately 3.5% of global cases, primarily affecting regions of South America, Southeast and South Asia, the Western Pacific, and Oceania (71). *Plasmodium* parasites display distinct developmental stages throughout their life cycle, including hepatic, blood, and gametocytic forms, which interact differently with host cells and can influence EV engineering strategy (3, 4). Species such as *P. vivax* and *P. ovale* also have an additional parasitic form, hypnozoites, characterized by a dormant state in the liver (72), which highlights the variety of proteins that could be target as well as strategies in choosing stages that could impact more in the infection progression.

An effective way of combating malaria includes strategies of prevention, not only protecting against mosquito bite, but also enhancing the immune response. In this regard, vaccines currently approved by the WHO, such as RTS,S, and R21, target the CSP protein present in sporozoites, responsible for initiating infection in the mammalian host (5, 73). However, the high antigenic variability of *Plasmodium* can reduce the efficacy of conventional treatments and vaccines. In this regard, vaccines based on EVs show promising potential.

**Figure 3:**
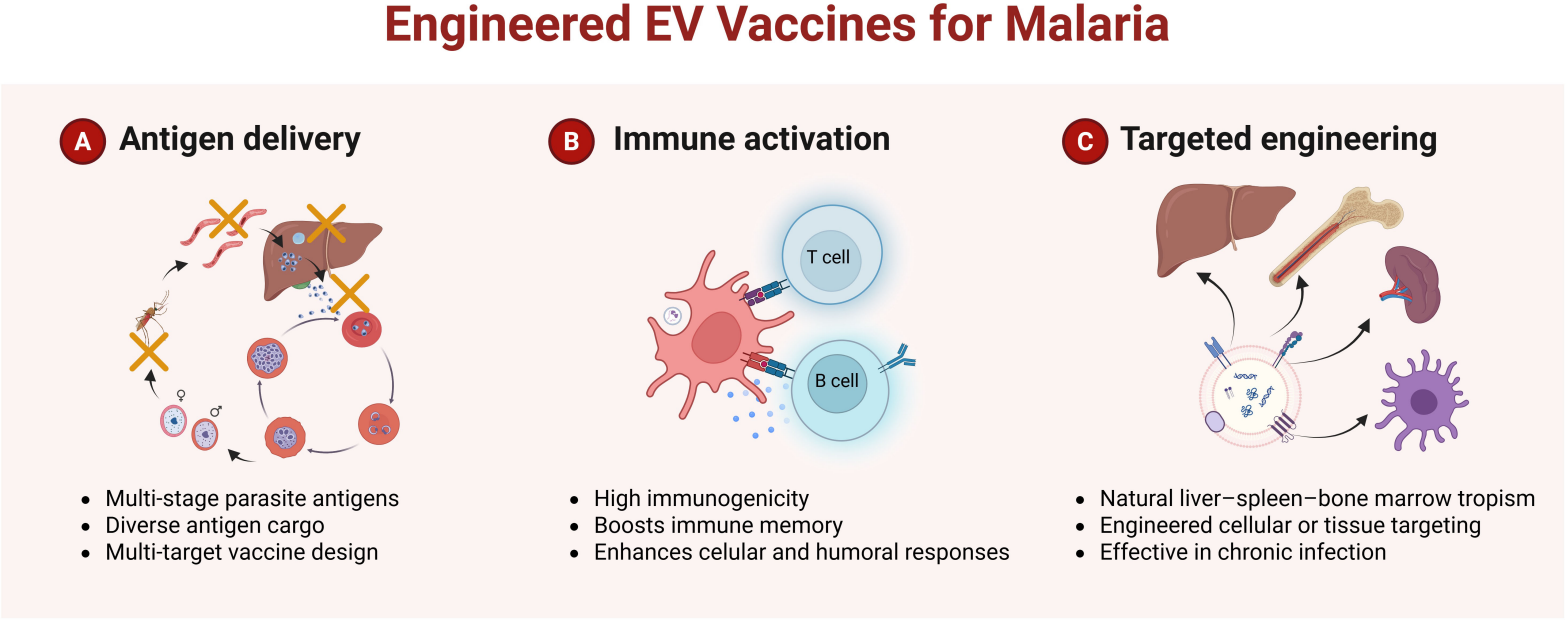
Advantages of EV-based vaccines for Malaria combat. There are four main ways in which EV-based vaccines can help combat malaria: **(a)** Antigen delivery: EVs can carry diverse molecular cargoes that target different stages of the parasite’s life cycle and help overcome antigenic diversity. **(b)** Immune activation: EV-based vaccines can stimulate robust immune responses, which is especially important for counteracting the parasite’s immune-evasion strategies. **(c)** Targeted engineering: EVs can be engineered to target multiple tissues, allowing vaccines to reach and act more effectively on organs that are particularly affected during infection. Created in BioRender. Albrecht, L. (2026). https://BioRender.com/f3yo1fx.

In the case of malaria, the delivery of vaccines using nanoparticles has intensified in recent years (74–79). According to studies, these molecules aid in the development of immunological memory and humoral response (**Figure 3**). To better contextualize the potential advantages and current limitations of EV-based vaccines in comparison with WHO-recommended malaria vaccines, **Table 2** summarizes key characteristics of RTS,S/AS01, R21/Matrix-M, and engineered EV platforms proposed here. This comparison highlights how EVs may expand antigen diversity, immune activation, and delivery possibilities, while also emphasizing the technological and regulatory challenges that still limit their clinical application.

**Table 2:** Comparison between approved malaria vaccines and potential EV-based vaccine.

Feature	RTS,S/AS01	R21/Matrix-M	EV-based vaccines (experimental platform)
Target antigen	CSP protein	CSP protein	Multiple antigens from different parasite stages
Vaccine platform	Recombinant protein-based vaccine with AS01 adjuvant	Recombinant protein-based vaccine with Matrix-M adjuvant	Engineered EVs carrying proteins, peptides, RNA, or DNA
Immunogenicity	Induces strong anti-CSP antibody responses with moderate cellular immunity	Higher anti-CSP antibody titers and high protective efficacy reported in clinical trials	Preclinical studies suggest the ability to induce both humoral and cellular immune responses
Durability of protection	Partial protection with antibody waning over time; booster doses required	Sustained efficacy observed after booster immunization, although antibody decline still occurs	Long-term durability remains unknown and requires clinical validation
Antigen flexibility	Restricted to CSP	Restricted to CSP	High flexibility for incorporation of multiple antigens
Ability to address antigenic variability	Limited by antigen specificity	Limited by antigen specificity	Potentially advantageous due to customizable multi-antigen loading
Manufacturing complexity	Established large-scale recombinant production	Recombinant production platform	Complex EV isolation, purification, characterization, and standardization processes
Storage requirements	Requires cold-chain storage	Requires cold-chain storage	Storage conditions depend on formulation; some EV preparations show increased stability in preclinical studies
Administration routes	Intramuscular	Intramuscular	Potentially adaptable to intramuscular, intranasal, or oral delivery
Clinical validation	WHO-recommended and implemented in endemic regions	WHO-recommended and under expanding implementation	Currently limited to experimental and preclinical studies
Main limitations	Moderate efficacy and waning immunity	Limited long-term real-world data	Lack of clinical studies, regulatory challenges, and limited scalability
Safety concerns	Well-characterized safety profile in large clinical trials	Favorable safety profile	Potential dual role depending on EV cargo and origin, requires rigorous safety characterization

The comparison reinforces that currently approved malaria vaccines remain restricted to CSP-based strategies, which may limit their effectiveness against the extensive antigenic diversity of *Plasmodium* parasites. In contrast, EV-based platforms offer greater flexibility for incorporating multiple antigens from different parasite stages, potentially enabling broader and more durable immune responses. Furthermore, EVs may stimulate both humoral and cellular immunity, including CD8+ T-cell activation, which remains relatively limited in current recombinant protein-based malaria vaccines. However, despite these theoretical and preclinical advantages, EV vaccines still face major challenges regarding manufacturing scalability, purification, regulatory standardization, and clinical validation.

Although there is not yet an extracellular vesicle (EV)-based malaria vaccine, it is assumed that it would be an efficient method, with high stability in the biological system, low rejection, and a high capacity to stimulate the immune system, being potentially more efficient than synthetic molecules (80). Furthermore, several studies suggest that EVs derived from *Plasmodium*-infected cells are promising as vaccine delivery systems, potentially increasing the efficacy and stimulation of immune system cells (81–83).

Few studies have investigated the role of EVs in malaria (84). For instance, exosomes derived from reticulocytes infected with *P. yoelii* can protect immunized mice against lethal infections, highlighting the potential of EVs carrying parasite antigens to confer protection (85). Conversely, EVs may also facilitate parasite adhesion, as shown by plasma EVs from *P. vivax*-infected patients signaling spleen fibroblasts (86). This dual functionality underscores the versatility of EVs, which can modulate host-parasite interactions in ways that may benefit either the host or the parasite, making them promising candidates for vaccine development.

Appropriate *in vivo* malaria models will also be essential to evaluate the efficacy, biodistribution, and safety of EV-based vaccines. Rodent malaria parasites such as *Plasmodium berghei* (87) and *P. yoelii* (88) represent valuable preclinical systems due to their well-established infection models and ability to reproduce hepatic and blood-stage immune responses in mice. In particular, *P. yoelii* models have already demonstrated the protective potential of parasite-derived EVs (85). In parallel, humanized mouse models infected with *Plasmodium* may provide an important translational platform to assess immune responses against human malaria antigens and to validate tissue-targeting strategies in a context closer to human infection (89–92). Together, these models may accelerate the optimization and preclinical validation of engineered EV vaccines for malaria.

Moreover, enhancing EVs through genetic engineering can substantially boost their ability to target specific cells and activate the immune system more effectively (22, 93). This ability might also help in chronic states of malaria, especially considering its dormant stage in illnesses caused by *P. vivax* and *P. ovale*. Beyond the apparent tropism of EV to the liver, bone marrow, and spleen (43), the EVs themselves can be engineered on purpose to such tissue-delivery.

Highlighting the EVs possibilities in real life conditions for combating malaria, a study demonstrated that EVs can be stored for up to 12 months at room temperature (94), facilitating vaccination logistics in endemic regions. Considering the children as a most fragile group, the intranasal route of administration has also proven effective, reducing the invasiveness of the procedure and maintaining stability for up to 3 months (50).

## Limitations

Despite the momentum behind EV technology, its application to malaria vaccines still faces significant hurdles. The field remains heavily reliant on data derived from cancer and viral models, leaving an important gap in malaria-specific research, while most available evidence is still restricted to preclinical studies, lacking the clinical validation required for translation into endemic settings. In addition, major technical challenges persist regarding large-scale manufacturing, purification, cargo standardization, and long-term storage stability. These limitations become even more pronounced when considering the remarkable antigenic diversity of Plasmodium parasites, which complicates the development of universally effective vaccine strategies. Another critical concern relates to the biological safety of EV-based approaches, since EVs may exert both protective and pathogenic effects depending on their cellular origin and molecular cargo. While parasite-derived EVs have been associated with protective immune responses (85), they may also contribute to parasite survival and cytoadherence. For instance, EVs from *P. vivax*-infected patients were shown to activate spleen fibroblasts through NF-κB signaling, potentially facilitating parasite sequestration and disease progression (86). These findings reinforce the importance of carefully controlling EV composition during vaccine development. In this context, engineering strategies may help minimize potential pro-parasitic effects by selectively enriching protective antigens, excluding immunopathogenic or adhesion-related molecules, and directing EV tropism toward professional antigen-presenting cells rather than tissues associated with parasite persistence. Therefore, rigorous safety profiling, functional characterization, and robust malaria-focused studies will be essential to determine the feasibility, safety, and long-term efficacy of engineered EV vaccines before their clinical implementation.

## Conclusions

EVs-based vaccines represent a versatile platform that unites targeted delivery, immune modulation, and antigen diversification in a single strategy. This is particularly valuable for malaria, where intense genetic variability generates new epitopes and undermines established targets. By reawakening protective responses and addressing both acute and chronic infections, EVs expand the possibilities of long-term disease control. Advances in engineering - including surface protein modification, signal peptide-guided targeting, and optimized cargo loading - strengthen their precision and efficacy. At the same time, improvements in stability and alternative administration routes enhance their feasibility in field conditions and resource-limited regions. Taken together, these innovations position engineered EVs as a transformative tool for next-generation vaccines, with the potential to reduce the burden of malaria and other neglected diseases worldwide.
